# Effect of Acetylation on the Nanofibril Formation of Chitosan from All-Atom De Novo Self-Assembly Simulations

**DOI:** 10.3390/molecules29030561

**Published:** 2024-01-23

**Authors:** Aarion Romany, Gregory F. Payne, Jana Shen

**Affiliations:** 1 Department of Pharmaceutical Sciences, University of Maryland School of Pharmacy, Baltimore, MD 21201, USA; akromany@umaryland.edu; 2 Institute for Bioscience and Biotechnology Research, University of Maryland, College Park, MD 20742, USA; gpayne@umd.edu

**Keywords:** polysaccharide, degree of acetylation, pattern of acetylation, molecular dynamics simulations, self-assembly

## Abstract

Chitosan-based materials have broad applications, from biotechnology to pharmaceutics. Recent experiments showed that the degree and pattern of acetylation along the chitosan chain modulate its biological and physicochemical properties; however, the molecular mechanism remains unknown. Here, we report, to the best of our knowledge, the first de novo all-atom molecular dynamics (MD) simulations to investigate chitosan’s self-assembly process at different degrees and patterns of acetylation. Simulations revealed that 10 mer chitosan chains with 50% acetylation in either block or alternating patterns associate to form ordered nanofibrils comprised of mainly antiparallel chains in agreement with the fiber diffraction data of deacetylated chitosan. Surprisingly, regardless of the acetylation pattern, the same intermolecular hydrogen bonds mediate fibril sheet formation while water-mediated interactions stabilize sheet–sheet stacking. Moreover, acetylated units are involved in forming strong intermolecular hydrogen bonds (NH–O6 and O6H–O7), which offers an explanation for the experimental observation that increased acetylation lowers chitosan’s solubility. Taken together, the present study provides atomic-level understanding the role of acetylation plays in modulating chitosan’s physiochemical properties, contributing to the rational design of chitosan-based materials with the ability to tune by its degree and pattern of acetylation. Additionally, we disseminate the improved molecular mechanics parameters that can be applied in MD studies to further understand chitosan-based materials.

## 1. Introduction

Chitosan is a linear copolymer of D-glucosamine (GlcN) and N-D-acetylglucosamine (GlcNAc) linked by β-1,4-glycosidic bonds. Owing to the solubility in aqueous solution, pH responsiveness, and antimicrobial properties [1], chitosan has found vast applications in biotechnology and pharmaceutics [2,3]. Chitosan is produced by deacetylation of chitin, nature’s second most abundant biopolymer, which is found in the shells of crustaceans and fungal cell walls [1]. Deacetylation is never complete, resulting in chains with varying distributions of acetyl groups or degrees of acetylation (DA) [4,5]. Additionally, depending on the method of deacetylation, the relative arrangement of GlcN and GlcNAc units or the pattern of acetylation (PA) may be different [4,5]. Generally, chitosan can be prepared chemically by treatment of chitin with a strong base which results in a random PA [4] or enzymatically via the action of chitin deacetylases which yields non-random PA [6,7].

**Figure 1:**
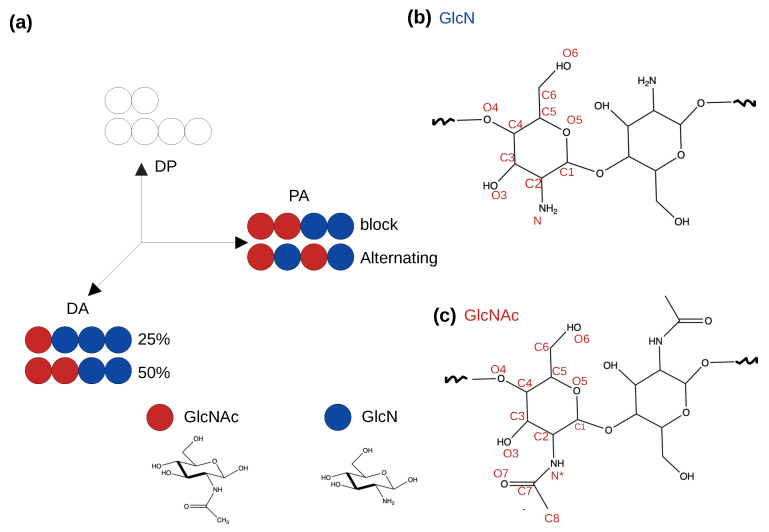
Schematics of a simplified “chitosan matrix” [5]. (**a**) Degree of polymerization (DP), degree of acetylation (DA) and pattern of acetylation (PA) describe the sequence of chitosan [5,8]. Blue and red circles represent the glucosamine (GlcN) and acetylglucosamine (GlcNAc) monomers, respectively. The effects of DA and PA are studied in this work. (**b**,**c**) Two GlcN or GlcNAc monomers connected by the glycosidic bond. Atom names are given.

In analogy to proteins, DA and PA along with the degree of polymerisation (DP) are the primary parameters that describe chitosan’s sequence [5,8] (Figure 1). Recent experimental work demonstrated that DP, DA, and PA influence the physicochemical properties and biological activities of chitosan [4,5,8,9]. However, the sequence–property or sequence–activity relationships of chitosan are poorly understood, which hinders the rational design and development of chitosan-based functional materials for novel applications [8]. Historically, this was a result of limited control in the synthesis of chitosan with specific DA, PA, and DP [5]. Improvements in synthesis and characterization methods [5] have allowed for the control of DA and DP, but control of PA remains a challenge [6,8].

Several experimental studies have examined the effect of DA on the physicochemical, material, and biological properties of chitosan. Foster et al. found that increasing DA within the range of 15–28% is associated with decreasing surface roughness, tensile strength, and crystallinity of chitosan films [10]. These results are consistent with an earlier study by Cao et al. [11], and may be related to the observation [12] that higher DA leads to lower cell adhesion and lower cell proliferation on chitosan films. More recently, Moerschbacher and coworkers examined the effect of PA on chitosan’s solution properties and gelation behavior using a variety of experimental techniques including ^13^C-NMR, rheology measurements, and circular dichroism spectroscopy [8]. They found that, in stark contrast to the commercially available random-PA chitosan, block-PA chitosan produced with chitin deacetylases had lower intrinsic viscosity in water, limited particle formation, and weak gelation behavior [8].

Despite the experimental efforts to investigate the sequence–property relationship of chitosan, very few computational or theoretical work has been published so far. Franca et al. [13] performed molecular dynamics (MD) simulations of a prebuilt chitosan nanoparticle (or nanofibril) comprised of nine (three by three matrix) antiparallel chains each having 10 monomer units in the block or alternating PA and different DAs. The results suggested that the chain solubility is inversely related to DA, and that chitosan nanofibril with block PA is more stable [13]; however, these conclusions were based on an extremely short (20 ns production run) MD trajectory of a minimally sized nanofibril. Importantly, the simulations started from a nanoparticle, which precludes the investigation of chitosan’s self-assembly behavior. More recently, Tsereteli and Grafmüller [14] developed a coarse-grained model for titratable chitosan and validated the model using Metropolis Monte Carlo simulations of a single chitosan chain with various DP (656–2100), DA (5–100%) and PA values at different solution pH conditions. Based on the radius of gyration of the single chitosan chain, study [14] suggested that increased DA leads to increased compaction or chain flexibility and a chain with alternating PA is more compact than the block-PA chain. Although these results are interesting and novel, the simulations did not account for chain–chain association, which is critical for understanding the sequence–property relationship of chitosan. Furthermore, due to the coarse-grained nature of the model, atomic details cannot be gleaned.

The objective of the present work is to understand at the atomic level the effects of DA and PA on chitosan’s self-assembled microscopic structure. MD simulations rely on accurate parameters in the molecular mechanics force field or energy function to describe the dynamics of molecules. Thus, we first refined the parameters in the current all-atom CHARMM molecular mechanics force field of chitosan [15,16]. We then conducted, to the best of our knowledge, the first all-atom de novo self-assembly MD simulations of chitosan chains with different DAs and PAs from the initially randomly distributed configurations in a solution. The simulations allowed us examination of the ways DA and PA influence the formation and the structure of the nanofibril. Qualitative comparison with the relevant experimental data is discussed. The present work provides an important step towards atomic-level understanding of how acetylation modulates chitosan’s physicochemical properties and contributes to the rational design of chitosan-based materials with tunable degree and pattern of acetylation. The improved force field parameters are disseminated to the community to facilitate future studies of chitosan-based materials.

## 2. Results and Discussion

### 2.1. Optimization of the Molecular Mechanics Force Field and Summary of Simulations

**Figure 2:**
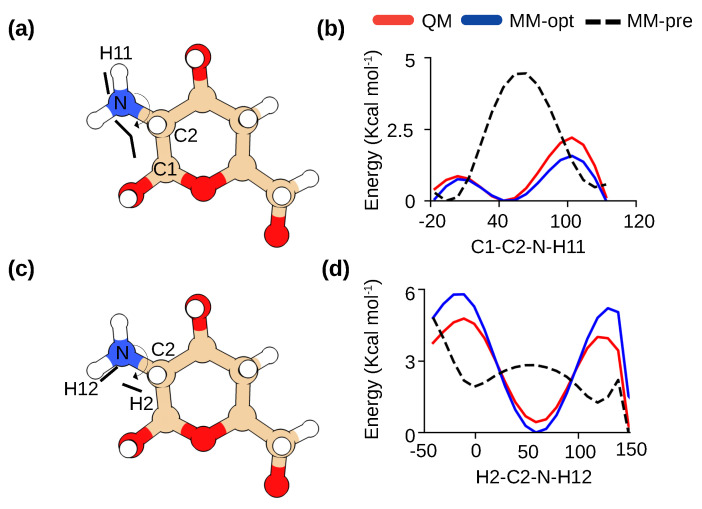
Optimization of the dihedral parameters for β-glucosamine in chitosan. (**a**,**b**) Definitions of C3–C2–N–HN1(HN2) and H2–C2–N–HN1(HN2) torsion angles in β-glucosamine. (**c**,**d**) Comparison of the potential energy scans for the two torsion angles calculated by quantum mechanics (QM) method (red) and CHARMM36 force field (before optimization in black and after optimization in blue). For QM calculations, the MP2/6-31G method was used following the protocol for CHARMM36 development [15,20].

The CHARMM36 carbohydrate force field [15,16] already contains optimized parameters for a subset of β-linked carbohydrates, including β-acetylglucosamine (GlcNAc), the parent molecule of β-glucosamine (GlcN). These two molecules differ only in two torsion angle parameters related to the primary amino group (Figure 1). In our previous studies of chitosan [17,18], aliphatic primary amine parameters were adopted according to the CGENFF force field [19]; however, we found that the potential energy profiles of the two torsional angles deviate significantly from the quantum mechanical (QM) calculations (Figure 2). Thus, following the protocol used in the development of the CHARMM36 carbohydrate force field [15,16], we manually adjusted the torsion parameters until they matched QM data (Figure 2b,d). All subsequent simulations were performed with these optimized parameters which are made freely available (see Data Availability).

Chitosan de novo self-assembly simulations were initiated with 24 chains randomly distributed in a 85 × 85 × 85 cubic box filled with water and periodically replicated in three dimensions to produce weight percent concentrations of 8.6% and 9.2% for the 20% and 50% DA systems, respectively. Each chain was comprised of 10 monomer GlcN or GlcNAc units, whereby the DA was 20% or 50% for all chains. At 50% DA, two different patterns of acetylation, alternating and block, were used. For each combination of DA or PA, triplicate runs were conducted starting from the same initial arrangements of the 24 chains but different initial velocities for atoms (velocity seeds). All trajectories were continued until the total number of interchain hydrogen bonds (h-bonds) and total solvent accessible surface area (SASA) plateaued, resulting in ∼2 μs for DA of 20% and ∼3–4.5 μs for DA of 50% runs (Table 1). We note that since the present work is focused on the effect of DA and PA on the self-assembled structure, the neutral glucosamine was used for the deacetylated units, i.e., high pH condition. Thus, the electrostatic repulsion between the protonated GlcN residues was not considered.

### 2.2. Chitosan Solubility and Self-Assembly Is Dependent on the Degree of Acetylation

We first investigated the effect of DA using simulations of 20% and 50% acetylated chitosan chains. We found that at 20% DA, no self-assembly occurred and the chitosan chains remained in solution after 2 μs, with only transient formation of intermolecular hydrogen bonds (h-bonds, Supplemental Figure S1). In contrast, at 50% DA, chain association via the formation of intermolecular h-bonds occurred rapidly after 1 μs of simulation (Supplemental Figure S2). This difference suggests that acetylation promotes chain–chain association, consistent with an early experimental study which showed that at above 50% DA, even protonated chitosan chains can aggregate [21]. We note that the observation that 20%-DA chitosan chains did not self-assemble within the simulation time reflects the short chain length (DP = 10). With a long chain length (or increased DP), chitosan chains can form a larger number of intermolecular h-bonds (see later discussion), and thus self-assembly can occur at lower DA, e.g., the nanofibrils of fully deacetylated 20 mer chitosan chains are stable (Romany and Shen, unpublished data). Thus, the effect of DA on self-assembly may be amplified in our simulations.

**Figure 3:**
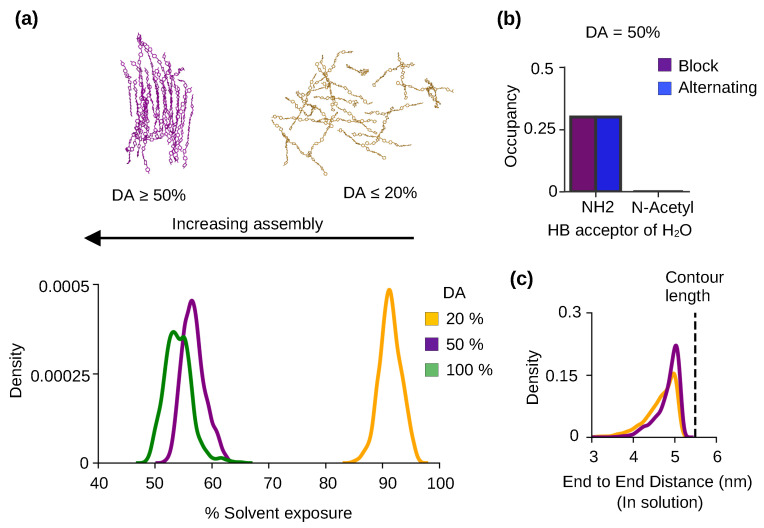
Chitosan solubility and self-assembly is modulated by the degree of acetylation. (**a**) The representative snapshots of 50% DA chitosan after 3 μs and 20% DA chitosan after 2 μs simulations. Probability distribution of the % solvent exposure for chitin (green), chitosan at 50% DA (purple) and 20% DA (yellow) with the block PA. Final 100 ns were used for calculations. Data for chitin (DA of 100%) were taken from our previous work [22]. (**b**) The average occupancy of the h-bond from water to the amino (NH2) or the acetylated amino (N-Acetyl) nitrogen per monomer unit for the block (purple) and alternating PA (blue) chitosan at 50% DA. For each trajectory frame, an occupancy of the h-bond was calculated by averaging over all 10 monomers in 24 chains. This occupancy was then averaged over the last 100 ns for all three trajectories. (**c**) Probability distribution of the end-to-end distance of 50% (purple) and 20% (brown) DA chitosan chains with block PA in solution. Only the chains in a solution were considered in the calculation. The end-to-end distance is defined as the distance between the center of mass of the second sugar unit and center of mass of the ninth sugar unit in a chitosan chain. The green dashed line represents the contour length (length in the built chitosan model).

To characterize solubility, we calculated the percent solvent exposure of the chitosan chains defined as the solvent accessible surface area (SASA) relative to that of a fully solvated chain. The data for chitosan chains with 20% and 50% DAs were compared. We also included a similar calculation of chitin, which is chitosan with 100% DA (taken from our previous study [22]). The probability distributions show that the % solvent exposure decreases with DA (peak of 90%, Figure 3a). At DA 50% and 100%, the percent solvent exposure per chain is below 60%, whereas at DA at 20% the solvent exposure per chain is near 100% (peak of 90%, Figure 3a). This can be explained, as water is excluded around the self-assembled chains with DA above 50%, whereas the unassembled chains with 20% DA are surrounded by a solvent.

To understand the physical basis for the effect of DA on the solubility and self-assembly of chitosan, we examined the h-bond formation between water and GlcNAc or GlcN. We found that the primary amine nitrogen of GlcN is able to accept h-bonds from water, but the acetylated nitrogen of GlcNAc does not form h-bonds with water (Figure 3b, purple bar). The same behavior is observed for chitosan chains with both block and alternating PAs (Figure 3b, purple and blue bars). Since lower-DA chitosan chains have higher amine nitrogen content compared to higher-DA chains, they form more h-bonds with water, which makes the solvated form energetically more stable. We suggest the difference in the h-bond formation with water as a major contributor to the solubility increase with decreasing DA. The amine–water h-bond formation is in direct competition with the chain–chain (NH–O6) h-bond formation which contributes to self-assembly. Thus, the h-bond formation between the primary amine and water also contributes to the decrease in self-assembly with decreased DA.

We next examined the effect of DA on the flexibility of chitosan chain by calculating the distribution of the end-to-end distances of the chains in a solution. For 20% DA chains, the distribution is slightly wider and the speak intensity is lower than that of 50% DA chains (Figure 3c), which indicates that lower-DA chains are more flexible compared to higher-DA chains in a solution. These data are consistent with the persistent length data from the experiments [21,23] but disagree with coarse-grained simulations which suggested that increased DA leads to increased chain flexibility [14]. These data support the notion that the bulkier acetamido groups (compared to the amino group) limit chitosan chain rotation such that chain stiffness increases with DA [21].

### 2.3. Pattern of Acetylation Influences Chitosan’s Self-Assembly Process

**Figure 4:**
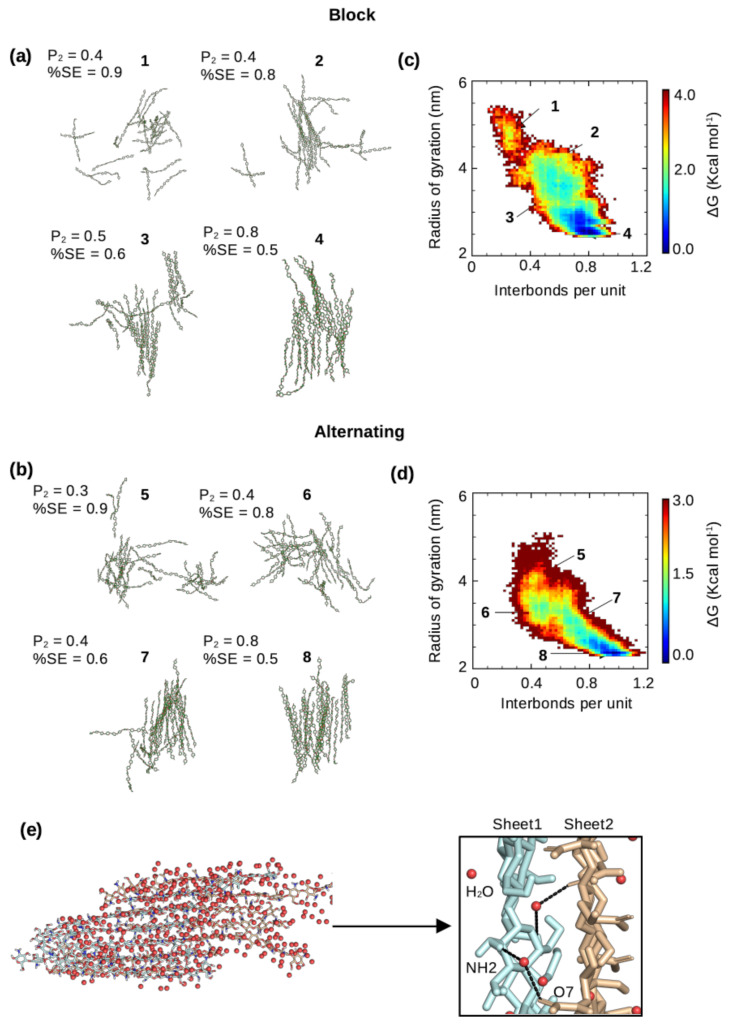
Dynamics of the self−assembly process of chitosan with block and alternating PAs. (**a**,**b**) Representative snapshots of chitosan with 50% DA and block (**a**) or alternating (**b**) PA along the local minima of the free energy surface (self-assembly pathway) shown in (**c**,**d**). Simulation Run 1 of the block PA and Run 3 of the alternating PA were used. The associated $P_{2}$-order parameters and average percent solvent exposure (relative to the fully solvated state) are given. (**c**,**d**) Free energy as a function of the radius of gyration and interchain h-bonds per monomer unit for chitosan with 50% DA and block (**c**) or alternating (**d**) PA. Only chains that formed part of the nanofibril were used for calculation. The runs with the highest number of interchain hydrogen bonding for both block (Run 1) and alternating (Run 3) PAs are shown here, while the rest are shown in Supplemental Figure S3. The $P_{2}$-order parameter was calculated using Equation (1), following our previous work [22,24]. (**e**) Snapshot of a nanofibril with water shown as red spheres. A zoomed-in view shows water-mediated interactions between two fibril sheets.

We examined and compared the self-assembly process of chitosan at 50% DA with the block and alternating PAs. Interestingly, regardless of PA, at the end of the simulation runs when the number of interchain h-bonds and solvent exposure plateaus, the chitosan chains formed a single nanofibril with at most two chains left in the solution (Figure 4a,b). Regardless of PA, the percentage of solvent exposure and the degree of order of self-assembled nanofibrils are also similar (Figure 4a,b). The latter was measured by the $P_{2}$-order parameter (Equation (1)) as in our previous work [22,24]. However, according to the number of interchain h-bonds and the total relative solvent exposure, the self-assembly process converged much faster (within ∼2 μs) for the block-PA chains in all three runs compared to all three runs of the alternating PA chains (more than 3 μs, Supplemental Figure S2). The recent experiment of Moerschbacher and coworkers demonstrated that the gelation kinetics of the biotech chitosan that contains block PA are distinct from those of the conventional, random-PA chitosan [8]. Specifically, they found that in contrast to random-PA chitosan, block-PA chitosan did not show a single critical gelation time [8]. Although the difference in the kinetics of self-assembly simulations cannot be directly compared to the difference observed in the experiment [8], they may be related. Moerschbacher and coworkers also found that block-PA chitosan nanoparticles showed a higher dispersity in size than random-PA nanoparticles, which was attributed to the formation of small aggregates [8]. The more rapid self-assembly of block-PA chitosan revealed by the simulations is consistent with this observation.

To quantify the self-assembly process, we used a low-dimensional free energy surface (FES) spanned by the radius of gyration ($R_{g}$) of all the chitosan chains and the number of interchain h-bonds per monomer unit (Figure 4c,d), following our previous work [22]. When the chitosan chains are in a solution (disassembled state), the radius of gyration of all chains is high and the number of interchain h-bonds is very low, while in the fully assembled state, the radius of gyration is low (the chains are associated) and the number of h-bonds is maximized. There are two extreme cases for the shape of the FES. A diagonally shaped FES suggests that the chain–chain hydrophobic association and h-bond formation are concomitant, whereas an L-shaped FES indicates that hydrophobic collapse proceeds h-bond formation [22]. Our recent simulations of chitin’s self-assembly process [22] demonstrated that chitin’s fibril formation can follow either a diagonally or an L-shaped mechanism. Here, the chitosan chains with both block and alternating PAs appear to more closely follow the diagonal mechanism, in which the hydrophobic association along with water expulsion occur concomitantly as the interchain h-bond formation (Figure 4c,d; Supplemental Figure S3). Interestingly, the free energy difference between the maximum (disassembled state) and global minimum (assembled state) is about 3 or 4 kcal/mol, similar to that found for the self-assembly of chitin [22]. It is interesting to note that, regardless of PA, the increase in the number of interchain h-bonds is correlated with the decrease in solvent exposure, consistent with the self-assembly simulations of chitin [22]. Nonetheless, the self-assembled chitosan nanofibrils with either block or alternating PA are hydrated, with sheet–sheet stacking stabilized by water-mediated interactions (Figure 4e), similar to the self-assembled chitin nanofibrils seen in our previous simulations [22].

### 2.4. The Self-Assembled Nanofibrils Are Comprised of Nearly Exclusively Antiparallel Chains Regardless of the Acetylation Pattern

Experimental studies of chitosan (presumably with a very low degree of acetylation) demonstrated several different crystalline polymorphs, with the hydrated forms (often referred to as tendon) being more abundant than the anhydrous forms (referred to as annealed), which are created by heating or exposure to carboxylic acid [25]. X-ray fiber diffraction measurements of the hydrated chitosan crystals [26,27] suggested that the fiber sheets are formed by antiparallel chitosan chains. Fiber sheets comprised of antiparallel chains were also suggested by the X-ray [28] and electron diffraction [29] studies of the anhydrous chitosan crystals prepared by high temperature. This arrangement was further confirmed by a subsequent study where the anhydrous chitosan crystals were prepared by using acetic acid [30] as well as a more recent high-resolution X-ray fiber diffraction measurement [25].

**Figure 5:**
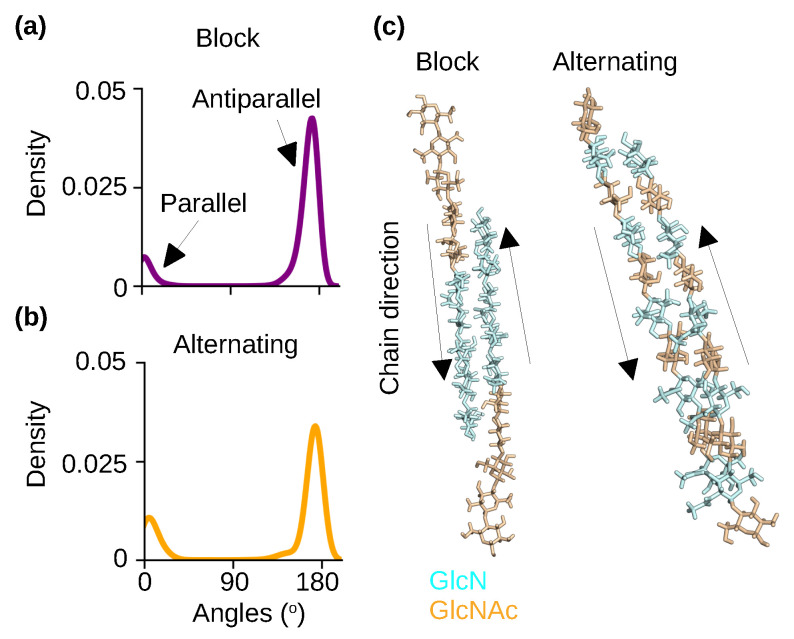
Chitosan chains are predominantly antiparallel in the self−assembled nanofibrils with either block or alternating PA. (**a**,**b**) Probability distribution of the angles between the adjacent chains for the chitosan with 50% DA and block (**a**) or alternating (**b**) PA. An angle around 0° indicates a parallel-chain alignment while an angle around 180° indicates an antiparallel-chain alignment. The final 100 ns of simulation time of each of the three trajectories was used for the calculation. (**c**) Representative snapshots of two neighboring chains in the nanofibrils with block and alternating PAs. The respective nanofibrils are displayed in Figure 4a,b.

To examine the effect of PA on chitosan’s polymorphism, we analyzed the relative orientations of chitosan chains in the self-assembled nanofibrils with block and alternating PAs. We note that the nanofibrils are hydrated. The chain orientation was characterized by calculating the angles between the adjacent chains. Angles of near 0° and 180° indicate parallel and antiparallel chains, respectively. The probability distributions of the angles show that antiparallel orientation is nearly exclusive for nanofibrils with either block or alternating PAs (Figure 5a–c), which is in agreement with the aforementioned X-ray diffraction results for hydrated chitosan crystals [25,28,29,30].

Importantly, the simulations of partially acetylated chitosan are in contrast to the recent simulations of the fully acetylated chitosan (chitin) [22], which resulted in nanofibrils comprised of both parallel and antiparallel chains, and the antiparallel orientation is more populated at high temperatures. In nature, chitin exists in three polymorphs: the most common α-chitin contains antiparallel chains [31], while β-chitin contains parallel chains [32] and the rare γ-chitin contains mixed antiparallel and parallel chains [33]. Thus, both our present and previous simulations [22] are consistent with the experimental observations, and together they suggest that deactylation promotes the antiparallel arrangement of chains.

### 2.5. Intermolecular Hydrogen Bonding Pattern Is Similar for the Nanofibrils with Block and Alternating PAs

**Figure 6:**
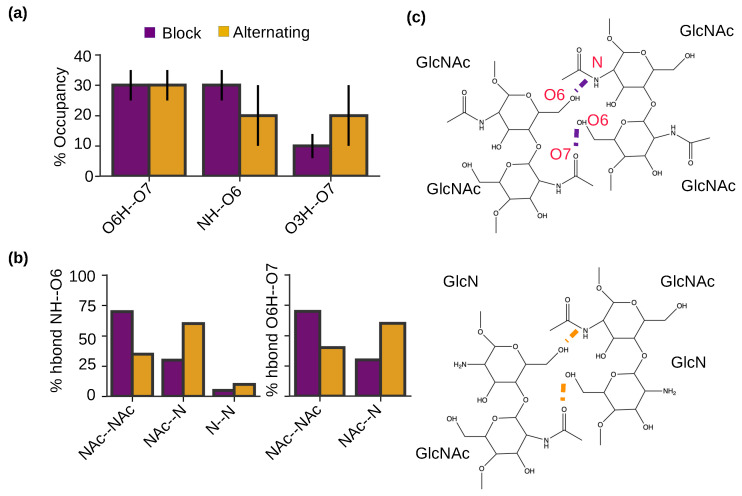
The intermolecular hydrogen bonding pattern is similar for the nanofibrils with block and alternating PAs. (**a**) Percentage occupancies of intermolecular h-bonds in self-assembled chitosan nanofibrils with 50% DA and block (purple) or alternating PA (brown). (**b**) Percentage of NH–O6 or O6H–O7 intermolecular h-bonds formed between two acetylated (NAc–NAc) units, between one acetylated and one deacetylated (NAc–N) units, and between two deacetylated units (N–N). The final 100 ns of simulation time in each of the three trajectories for each PA was used for calculation. Only chains aligned antiparallel to each other were considered. (**c**) Two-dimensional structure showing the intermolecular h-bonds between two acetylated units (**top**) and between one acetylated and one deacetylated units (**bottom**).

To examine the intermolecular h-bonds that drive the self-assembly of chitosan and the potential effect of PA, we calculated the occupancies of specific h-bonds formed between the antiparallel chains for the nanofibrils with block and alternating PAs (Figure 6a). Interestingly, the nanofibrils with both PAs show the same intermolecular h-bonds, between O6H and O7, between NH and O6, and between O3H and O7 (Figure 6a). This is consistent with our previous simulations of chitin [22], which found the same three h-bonds in the self-assembled chitin nanofibrils comprised of antiparallel chains, whereas a different NH–O7 h-bond was formed between the parallel chains. It is worthwhile noticing that the O6H–O7 and NH–O6 h-bonds are dominant in the chitosan nanofibrils with block PA (Figure 6a), which is consistent with the chitin nanofibrils of antiparallel alignment [22]. In contrast, the O6H–O7 h-bond dominates the chitosan nanofibrils with alternating PA (Figure 6a). This analysis suggests that chitosan nanofibrils with block PA are more similar to the chitin nanofibrils with antiparallel alignment (i.e., α-chitin). X-ray or electron diffraction measurements of both hydrated [26,27] and anhydrous chitosan crystals [26,27] suggested that the chains on the fibril sheets are antiparallel and they are joined together by NH–O6 h-bonds. Considering that NH–O6 h-bonds have a higher relative occupancy in the simulated block-PA vs. alternating-PA nanofibrils, we suggest that block-PA chitosan is more similar to fully deacetylated chitosan.

To further understand how PA influences the h-bonding pattern of self-assembled chitosan nanofibrils, we examined the formation of the two dominant intermolecular h-bonds for blockNH–O6 and O6H–O7 h-bonds between two acetylated (GlcNAc–GlcNAc), two deacetylated (GlcN–GlcN), and mixed acetylated and deacetylated (GlcNAc–GlcN) glucosamine units (Figure 6b,c). In the nanofibrils with block PA, the majority (70%) of NH–O6 h-bonds are between GlcNAc and GlcNAc, followed by 25% between GlcNAc and GlcN and 5% between GlcN and GlcN units (Figure 6b left and Figure 6c, respectively). The finding that the h-bonds prefer to form between acetylated units was initially surprising, as it is unlikely for in-register antiparallel chitosan chains with block PA. However, it is consistent with our observation that chitosan chains with block PA tend to be off register in nanofibrils (Figure 5c). In contrast and as expected, for nanofibrils with alternating PA, the majority (∼60%) of NH–O6 h-bonds are formed between GlcNAc and GlcN units. Nonetheless, 30% of NH–O6 h-bonds are formed between GlcNAc and GlcNAc and 10% between GlcN and GlcN units, which can be partly attributed to the slightly off-register alignment between the two antiparallel chains in the nanofibrils with alternating PA (Figure 5c). Similar to intermolecular NH–O6 h-bonds, intermolecular O6H–O7 h-bonds also prefer to form between GlcNAc and GlcNAc units in the nanofibrils with block PA and between GlcNAc and GlcN units in the nanofibrils with alternating PA (Figure 6b right and Figure 6c, respectively). Taken together, the analysis of h-bonding suggests that glucosamine acetylation plays a central role in stabilizing nanofibril formation. We note that with longer chains (i.e., higher DP), the occupancies of intermolecular h-bonds, including the NH–O6 h-bonds between two GlcN groups, are expected to be much higher due to the more stable nanofibril formation (Romany and Shen, unpublished data); however, it would not affect our conclusion that acetylation strengthens the NH–O6 h-bonds, which is related to the increased solvation of amino vs. acetamide groups (Figure 3b).

## 3. Concluding Discussion

MD simulations employing the newly optimized force field parameters for β-glucosamine were conducted de novo to investigate the self-assembly of 10 mer chitosan chains with different DAs and PAs. Consistent with the experimental solubility data [21], MD simulations showed that the solvent accessibility of chitosan chains decreases with increasing DA. At 50% DA and with either block or alternating PA, the chitosan chains associated to form hydrated nanofibrils comprised nearly exclusively antiparallel chains, in agreement with the fiber diffraction measurements of the hydrated chitosan crystals [26,27]. Since our previous simulations and experiments [31] demonstrated that chitin nanofibrils can display both antiparallel and parallel chain arrangements, the new simulation results suggest that acetylation promotes parallel chain arrangements.

Further analysis of the self-assembled nanofibrils of 50% DA chitosan found that nanofibrils of block and alternating PAs share three common intermolecular h-bonds, O6H–O7, NH–O6, and O3H–O6, with the first two being most stable. Interestingly, for block PA, intermolecular h-bonds mostly occur between two acetylated units, which was initially surprising given the antiparallel chain arrangements; however, this can be rationalized by the off-register alignment of two antiparallel chains with block PA. In contrast, for the alternating pattern, intermolecular h-bonds most frequently occur between one deacetylated and one acetylated units, which is expected from the acetylation pattern. Another related finding from MD simulations is that the NH–O6 h-bond between two deacetylated units is significantly weaker than between two acetylated units or between one acetylated and one deacetylated units. This difference suggests that acetylation promotes self-assembly, which is consistent with the decreased solubility of chitosan with increased DA [21]. The latter can be attributed to the decreased solvation of acetamide relative to (neutral) amino group.

The main caveat of the current study is the minimalist nature of the model system, i.e., 24 very short (10 mer) chitosan chains (about 100 times shorter than the experimental chitosan chains). With very short chains, the chain–chain association is significantly weakened relative to the longer chains due to the smaller number of intermolecular h-bonds. For example, the NH–O6 h-bonds are unstable here but very stable between the 20 mer chitosan chains (Romany and Shen, unpublished data). We anticipate that the self-assembly simulations of longer chains and a larger number of chains would produce more intermediate states (local free energy minima) than the 24 10 mer chain simulations. This is because the 10 mer chain length is close to the persistence length [18], and as such, the conformational effects due to chain bending or the entanglement effects due to interactions of many chains are largely neglected. The use of a minimalist model system is due to the constraint of computational capabilities. In our simulations, the self-assembly of 24 10 mer chitosan chains occurred on the order of microseconds, which is more than seven orders of magnitude faster than the experimental observation of chitosan’s hydrogel formation resulting from chains that are 100 times longer. Self-assembly simulation of chains with much higher degrees of polymerization is computationally prohibitive. Due to the limited sampling time, the order the self-assembled nanofibrils observed in our simulations may be underestimated. Nonetheless, the comparison between block-PA and alternating-PA nanofibrils is robust given similar simulation time and multiple trajectories. Taken together, our simulations provide insights into the dynamics and mechanisms of self-assembly of chitosan that are difficult to observe by experiments. The analysis of the nanofibril structures and h-bonding patterns deepens the understanding of how PA influences chitosan’s physicochemical properties. We demonstrated that regardless of PA, chitosan’s remarkable self-assembly is still observed. With the growing demand for biodegradable and sustainable materials, understanding the sequence–structure relationship is a key to the rational design of novel chitosan-based materials.

## 4. Materials and Methods

### 4.1. Force Field Parameterization

The starting parameters for GlcN were based on the sugar parameters available for GlcNAc (β(1-4)acetyl-glucosamine) and modified CGenFF methyl group parameters [19]. The parameters for two dihedral angles related to the amine group, C1–C2–N–H11 and H2–C2–N–H12, were optimized (Figure 2). Staring from the diffraction structure of Okuyama and Ogawa [26], the model compound (Figure 2) first underwent geometry optimization using quantum mechanical (QM) calculations at the MP2 level of theory and 6-31G(d) basis set. The QM potential energy surface (PES) for H2–C2–N–H12 and C1–C2–N–H11 were then calculated at the MP2 level of theory and 6-31G(d) basis set. The parameters were manually tuned until QM PES and molecular mechanics (MM) PES were in good agreement. All QM calculations were performed using Gaussian-03, Revision C.02 [34]. Parameterization was facilitated using FFparam software [35].

### 4.2. Molecular Dynamics Simulation

All-atom molecular dynamics (MD) simulations were performed using the AMBER20 program [36]. Chitosan chains were represented by the CHARMM36 carbohydrate force field [15,16] and the modified CHARMM General force field parameters [19] (see Section 2.1). Different acetylation patterns and degrees of acetylation were produced by modifying a 10 mer chitin chain using Avogadro molecular editor [37]. For each distinct system, PACKMOL [38] was used to randomly distribute 24 chains in a 85 × 85 × 85 cubic box solvated with CHARMM-style TIP3P water model [39]. Simulation systems comprised approximately 92,493 atoms and the weight percentage of chitosan was 8.6% for 20% DA and 92,493 atoms with a weight percentage of 9.2% for 50% DA.

A total of 9 simulations were conducted of the system. Three independent simulations were performed for each system starting from different random velocity seeds. The systems first underwent energy minimization with a harmonic restraint potential (force constant of 10 kcal/mol/A) placed on heavy atom positions. The system was then heated over 1 ns to 300 K in the NVT ensemble. The system was then equilibrated for a total of 100 ns in which harmonic potential force constant was gradually reduced from 5, 2.5, 1, 0.1 to 0 kcal/mol/A under constant NPT conditions. The temperature was maintained using a Langevin thermostat [40] and the pressure was maintained at 1 bar using the Monte Carlo barostat [41]. Each production run lasted between 3 μs and 4.5 μs under the constant NPT condition (Table 1). van der Waals interactions were smoothly switched to zero from 10 to 12 A. The particle mesh Ewald method was used to calculate long-range electrostatic energies with a sixth-order interpolation and 1 Å grid spacing. Bonds involving hydrogens were constrained using the SHAKE algorithm [42] which allows the use of a 2 fs time step for integration. Subsequent analysis of simulations was performed using cpptraj [43] and VMD [44].

### 4.3. Data Analysis

All trajectory analysis was performed with the CPPTraj program [43]. The P_2_-order parameter was defined as [24]

(1) $$P_{2}=\frac{1}{N}\sum_{i=1}^{N}{\left({\vec{v}}_{i}.\vec{d}\right)}^{2},$$

where ${\vec{v}}_{i}$ is the normalized molecular vector connecting the center of mass of the second and ninth sugar rings in an individual chain. $\vec{d}$ is the director, which is a normalized average vector of the unsigned orientations of all individual ${\vec{v}}_{i}$ and *N* is the number of molecular vectors (number of chitosan chains). P_2_ calculation was performed with the CPPTraj program [43] and in-house Python scripts. SASA calculation was based on the LCPO algorithm of Weiser et al. [45].

## Figures and Tables

**Table 1:** Summary of de novo self-assembly MD simulations of chitosan chains in water.

DA	PA	DP	Chains	Simulation Time
20%	Block	10	24	3 × 2 μs
50%	Block	10	24	3 × 3 μs
50%	Alternating	10	24	2 × 4 μs, 4.5 μs

## Data Availability

All simulation input files, analysis scripts, and optimized force field parameters are freely available for download at https://github.com/janashenlab/chitosan (accessed on 14 January 2024).

## Supplementary Materials

The following supporting information can be downloaded at: https://www.mdpi.com/article/10.3390/molecules29030561/s1, Figure S1: Number of interchain hydrogen bonds per monomer unit and percent solvent exposure of all chitosan chains as a function of simulation time with 20% DA and block PA. Figure S2: Number of interchain hydrogen bonds per monomer unit and percent solvent exposure of all chitosan chains as a function of simulation time with 50% DA and block or alternating PA. Figure S3: Free energy as a function of the radius of gyration and intermolecular hbonds per monomer unit from the self-assembly simulations of 50% DA chitosan.
