# Supplementary material for: Effect of Acetylation on the Nanofibril Formation of Chitosan from All-Atom De Novo Self-Assembly Simulations
Source: Molecules. 2024 Jan 23;29(3):561. doi: 10.3390/molecules29030561 (PMC10856132; doi:10.3390/molecules29030561)
Supplement: Supplementary file 1 [file molecules-29-00561-s001.zip › molecules-2802209-supplementary.pdf]

Supporting Information: Effect of Acetylation on the Nanofibril Formation of Chitosan From All-Atom De Novo Self-Assembly Simulations

Aarion Romany,[†] Gregory F. Payne,[‡] and Jana Shen[*,†]

† *Department of Pharmaceutical Sciences, University of Maryland School of Pharmacy, Baltimore, MD 21201*

‡ *Institute for Bioscience and Biotechnology Research, University of Maryland, College Park, MD 20742*

E-mail: jana.shen@rx.umaryland.edu

List of Figures

S1 Time series of the interchain hydrogen bond formation of chitosan chains at 20% DA S-3

S2 Time series of the interchain hydrogen bond formation of chitosan chains at 50% and alternating pattern S-4

S3 Free energy as a function of the radius of gyration and intermolecular hbonds per monomer unit S-5

Supplemental figures

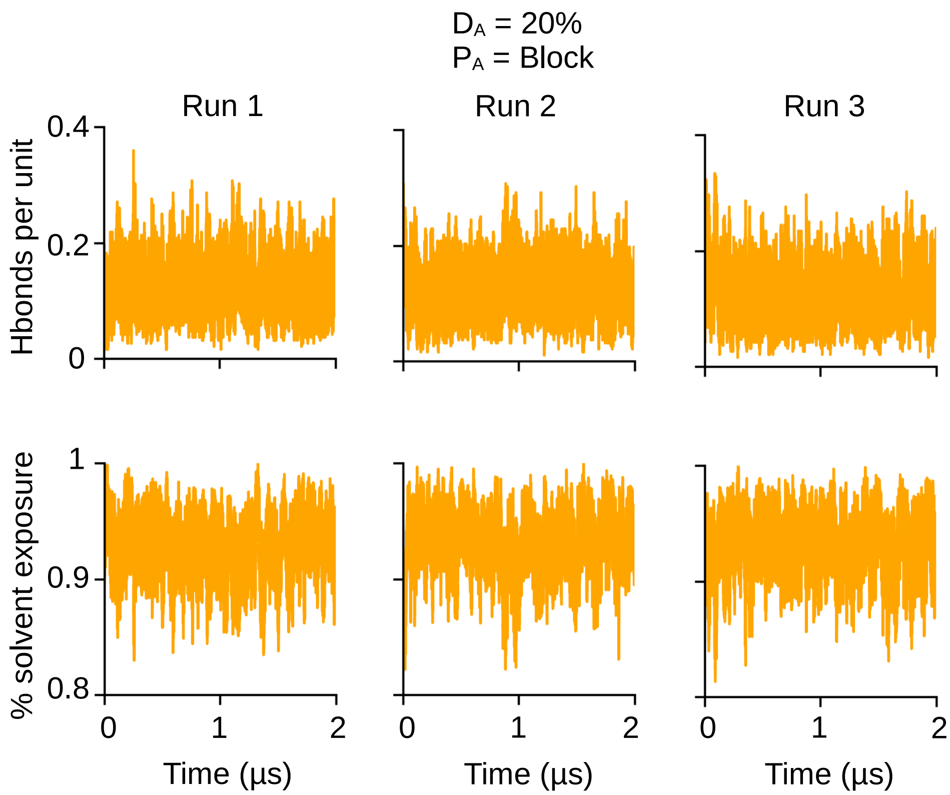

Figure S1: **Number of interchain hydrogen bonds per monomer unit and percent solvent exposure as a function of simulation time for chitosan at 20% DA with block PA.** A hydrogen bond is considered present if the heavy-atom donor-acceptor distance is below 3.5 Å and the donor-hydrogen-acceptor angle greater than 135°. Note, all 24 chains were used at each temperature. % solvent exposure is calculated as the solvent accessibility surface area (SASA) divided by the SASA of a fully solvent exposed chain.

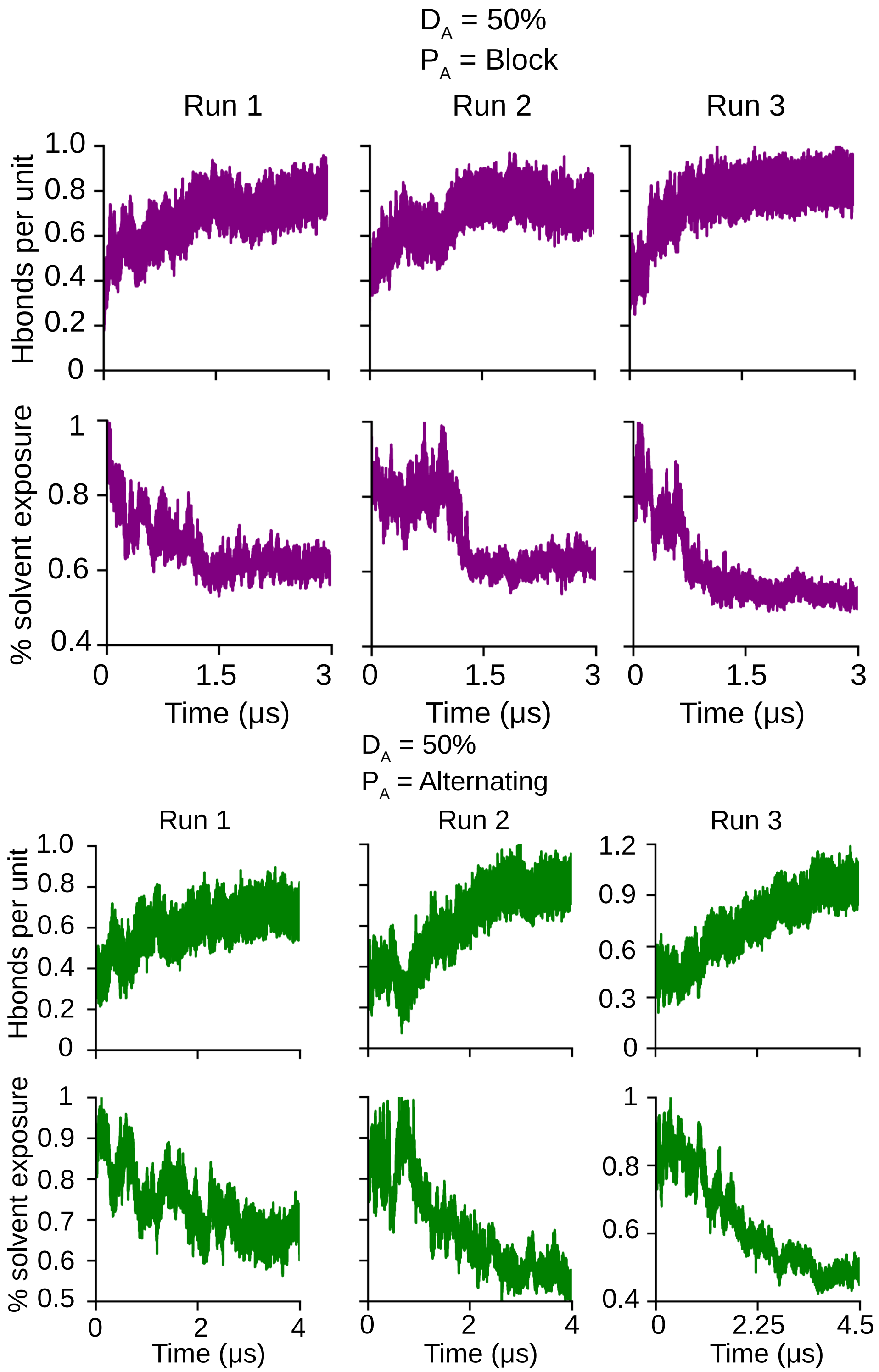

Figure S2: **Number of interchain hydrogen bonds per monomer unit and percent solvent exposure of all chitosan chains as a function of simulation time with 50% DA and block or alternating PA.** The data from the three independent runs of the block and alternating PA chitosan are shown in the top and bottom panels, respectively.

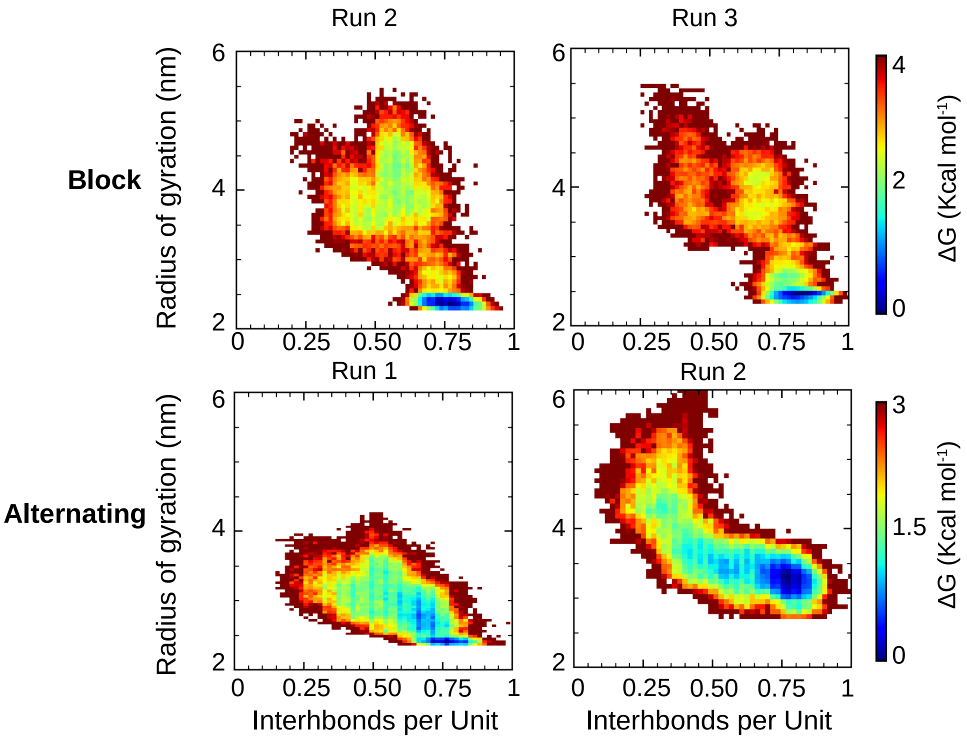

Figure S3: **Free energy as a function of the radius of gyration and intermolecular hbonds per monomer unit from the self-assembly simulations of 50% DA chitosan.** The two independent runs of the block (top) and alternating (bottom) PA chitosan are shown. The third run for bo are given in the main text. The entire simulation length was used in this calculation for each run.
